# Full‐Polarimetric Synthesized Holographic Displaying Empowered by Chirality‐Assisted Metasurface

**DOI:** 10.1002/smsc.202400138

**Published:** 2024-06-21

**Authors:** Yueyi Yuan, Wenjie Zhou, He Zhang, Yuxiang Wang, Hua Zong, Yue Wang, Yongkang Dong, Shah Nawaz Burokur, Kuang Zhang

**Affiliations:** ^1^ Harbin Institute of Technology No. 92 Xidazhi Street Nangang Distinct Harbin Heilongjiang Province 150001 China; ^2^ Xi'an University of Technology No. 5 Jinhua South Road Beilin District Xi'an Shaanxi Province 710048 China; ^3^ LEME Univ Paris Nanterre F92410 Ville d’Avray France

**Keywords:** chirality‐assisted phase, circular polarization, full‐polarimetric synthesize, holographic displaying, metasurface

## Abstract

Metasurface‐based holography provides tremendous advances in multi‐dimensional detection, super‐resolution imaging, and cryptography applications. Current mainstream researches on holographic metasurface are primarily focused on exploring degrees of freedom to enhance information multiplexing capability. Nevertheless, from the information security point of view, it is necessary to integrate existing available freedom resources, such as multi‐polarization components, to enhance the security of holographic encryption. Herein, a full‐polarimetric synthetization scheme is proposed for holographic displaying to develop a novel approach for information and imaging encryption. By exploiting chirality‐assisted metasurface as the implementation platform, quadruplex circular polarization components are independently phase‐modulated with separate holographic sub‐imaging. For an intuitive demonstration, linear polarization is set as the encoded state to acquire the synthesized intensity image with “HIT” characters. Hence, the output holographic information in transmission field can be successfully distinguished with the valid polarization. Additionally, the sensitivity and robustness property of the synthesized holographic performance is experimentally evaluated against ergodic elliptical polarization states, where the optimal performance of working efficiency and signal‐to‐noise ratio only appear under the preset linear polarizations. These results effectively prove the feasibility of the polarization integration hologram, opening the door to novel solutions for future full‐polarimetric encryption strategies.

## Graphical abstract

A novel approach of full‐polarimetric holographic displaying enabled by chirality‐assisted metasurface is proposed. By contrast to traditional polarization‐multiplexing hologram metasurface, quadruplex circular polarization components are synchronously and independently phase‐modulated with separate sub‐imaging. Thus, integral holographic image is distinguished with optimal high performance in terms of efficiency and signal‐to‐noise ratio only under the proper cipher polarization state.

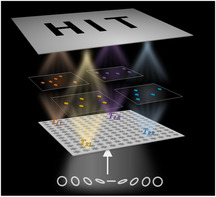

## 1 Introduction

Holographic display,^[^ ^1^ ^]^ relying on the spatial interference between the objective and reference intensity and phase information, has been acting an indispensable role in modern encryption techniques,^[^ ^2^, ^3^ ^]^ cryptography,^[^ ^4^ ^]^ microscopy,^[^ ^5^ ^]^ and metrology,^[^ ^6^ ^]^ and so forth.^[^ ^7^ ^]^ Conventional methods for holography depend on spatial light modulators, complex optical system constituting of waveplates and gratings, which suffer from limitations in practical applications, such as limited resolutions and field‐of‐views, bulk volumes, and narrow bandwidth.^[^ ^8^, ^9^, ^10^ ^]^ With the emergence of metasurfaces, composed by numerous elaborated meta‐atoms in sub‐wavelength scale, artificial manipulation of electromagnetic (EM) waves meets the compact and intuitive implementation platform requirements and inaugurates an unprecedented period of development.^[^ ^11^ ^]^ Metasurfaces can be constructed not only for optimizing the performances of classical optical devices^[^ ^8^, ^12^ ^]^ but also producing unprecedent functionalities that traditional devices are unable to achieve.^[^ ^13^, ^14^, ^15^ ^]^ As such, metasurfaces have been widely applied in optic cameras,^[^ ^16^, ^17^ ^]^ wireless communications,^[^ ^18^ ^]^ spectrum detections,^[^ ^19^ ^]^ multi‐dimensional imaging,^[^ ^20^ ^]^ and so forth.^[^ ^21^, ^22^ ^]^ Particularly, metasurface‐based holography has experienced rapid developing stages, from the single holographic imaging with enhanced quality to versatile hologram multiplexing, to satisfy the increasing requirements for information security and high‐level encryption.^[^ ^23^, ^24^, ^25^ ^]^

Due to the precise and flexible modulation abilities of metasurface, more than single available channels for holographic multiplexing have been explored by simultaneously utilizing several intrinsic EM attributes including but not limited to the amplitude, phase, wavelength, incident angles, and angular momentum.^[^ ^26^, ^27^ ^]^ Herein, phase‐engineered metasurfaces constitute the initial and basic scheme for holography ranging from microwave to optical regimes, which are widely studied for hologram imaging decoupling and integrations.^[^ ^28^, ^29^ ^]^ When the amplitude is set as the hologram reconstruction condition, near‐field nano‐printing, and gray‐scale imaging based on Malu's law are widely explored by dielectric nano‐antennas.^[^ ^30^ ^]^ Complex amplitude can be regarded as an effective factor in improving the imaging resolution and reconstruction quality.^[^ ^31^, ^32^ ^]^ For wavelength multiplexing, full‐color holographic displaying based on multi‐tasked metasurfaces is considered in the visible spectrum.^[^ ^33^, ^34^ ^]^ Incident angle can also be utilized as a design factor to achieve near‐field imaging and displaying.^[^ ^35^, ^36^ ^]^ Orbital angular momentum can be regarded as a high‐dimensional physical degree for holographic information carriers multiplexing based on its theoretically infinite orthogonal modes.^[^ ^37^, ^38^ ^]^ To further develop multiplexing channels, noise has been creatively employed to breakthrough the conventional polarization channel limit of birefringent metasurfaces.^[^ ^39^ ^]^ Besides, polarization state, as the most common and extensively applied EM property,^[^ ^40^ ^]^ owns unique advantages for holographic displaying, not only on account of its abundant parametric information of target objects but also due to its compatibility with all aforementioned EM attributes.^[^ ^41^, ^42^ ^]^ On this basis, from the perspective of information security, channel multiplexing is the precondition for holographic encryption, as the significant basis for imaging cryptography.^[^ ^43^, ^44^ ^]^ The current metasurface‐based platforms for holographic encryption mainly concentrate on exploring inexhaustible multiplexing channels and directly imposing the target information in one of them.^[^ ^20^, ^45^, ^46^, ^47^ ^]^ Nevertheless, in addition to digging extra channels, how to synchronously exploit multiple degree components to co‐establish the target ciphertext, would be a crucial and advanced strategy to enhance the information security by adopting intricate decryption key for cryptography and holography.

**Figure 1:**
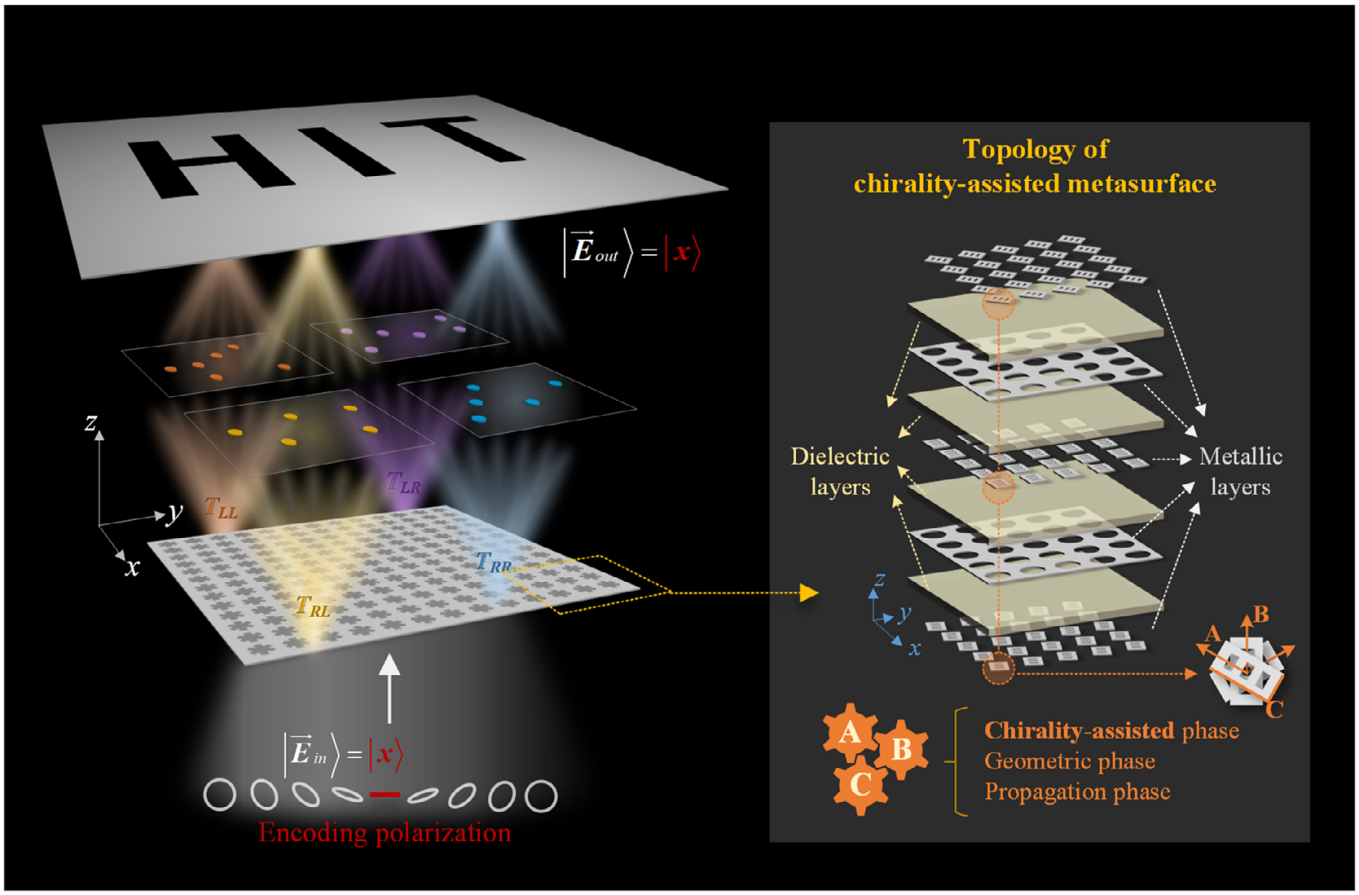
Schematic illustration of the proposed full‐polarimetric synthetization of hologram displaying. Metasurface with stacking and twisted multilayered structure is exploited to simultaneously phase‐modulate quadruplex circular polarization transmission components, while imposing independent sub‐imaging to synthesize the integral holographic “HIT” displaying. Under arbitrary elliptical polarization states illumination, the optimal performances of target “HIT” image can only be observed with the cipher linear polarization. The inset shows topological schematic of chirality‐assisted metasurface configuration with five metallic and four dielectric layers.

In this article, to achieve the synthesized high‐security holographic displaying, we demonstrate a universal scheme of full‐polarimetric wavefronts integration by utilizing quadruplex circular polarization components, which is implemented by twisted‐stacking metasurface configuration with chirality‐assisted, propagation and geometric phases, as conceptually illustrated in **Figure** **1**. Under circular polarization basic transmission matrix, full quadruplex polarization components are independently phase‐modulated by twisted‐stacking metasurface configuration. With the experimental verification, we construct a multilayered metasurface lens to separately impose segmental imaging in full quadruplex polarization channels, and then generate an integral holographic displaying under a fixed incident polarization. Additionally, we evaluate the robustness and sensitivity of holographic performances against full‐stokes incident polarization states within 10% relative bandwidth, where the measured working efficiency exceeds 90% and signal‐to‐noise (SNR) can reach 8 dB around operation imaging frequency. In contrast to traditional multiple polarization channel expansion, we integrate full polarization components under circular polarization basis to achieve a synthesized holographic displaying scheme. The outcome presents great potentials in enhancing the security of cryptographic strategies, and prospects in tridimensional holographic detection and communication systems.

## 2 Operation Principles and Implementation

### 2.1 Theoretical Principles of Full‐Polarimetric Synthetization

The target polarimetric synthetization of holographic displaying scheme can be simplistically represented by an artificial superposition of full mutually orthogonal polarization components, where the available amplitude and phase responses can be synchronously tailored. Considering the orthogonality and robustness of left‐ and right‐hand circular polarization (LHCP and RHCP) states, here we directly take advantages of circular polarization basis transmission matrix $T_{\text{cir}}=\left[\begin{array}{cc} T_{\text{LL}} & T_{\text{LR}}\\ T_{\text{RL}} & T_{\text{RR}} \end{array}\right]$, where each complex coefficient denotes an independent polarization component and the subscripts represent LHCP or RHCP state of output and input. Firstly, we start theoretically from the interaction effects of EM wave passing through the metasurface model. Accompanied with polarization preservation and conversion, this transmission process can be expressed as:

(1) $$T_{\text{cir}}\left|L\right\rangle =\kappa_{1}\left(\left|T_{\text{LL}}\right|\right)e^{i\cdot \Phi_{1}\left(∠T_{\text{LL}}\right)}\left|L\right\rangle +\kappa_{2}\left(\left|T_{\text{RL}}\right|\right)e^{i\cdot \Phi_{2}\left(∠T_{\text{RL}}\right)}\left|R\right\rangle$$

(2) $$T_{\text{cir}}\left|R\right\rangle =\kappa_{3}\left(\left|T_{\text{LR}}\right|\right)e^{i\cdot \Phi_{3}\left(∠T_{\text{LR}}\right)}\left|L\right\rangle +\kappa_{4}\left(\left|T_{\text{RR}}\right|\right)e^{i\cdot \Phi_{4}\left(∠T_{\text{RR}}\right)}\left|R\right\rangle$$

where *κ*_*i*_ (*i* = 1, 2, 3, 4) is the introduced amplitude weight coefficient of four distinct complex transmission coefficients *T*_LL_, *T*_LR_, *T*_RL_, and *T*_RR_, “| |”, “∠” are the amplitude and phase responses of these polarization preservation and conversion processes, |L⟩=|1,0⟩ and |R⟩=|0,1⟩ in circular polarization base.

Attributed to the orthogonality between these full polarization components, far‐field interferences would result in the complete holographic information displaying under a specific input‐ output polarization transformation process $\left|{\vec{E}}_{\text{in}}\right\rangle \to \left|{\vec{E}}_{\text{out}}\right\rangle$, which can be further analyzed through Rayleigh–Sommerfeld theory:

(3) $$|{\vec{E}}_{\text{out}}\rangle =T_{\text{cir}}|{\vec{E}}_{\text{in}}\rangle \approx \sum_{i=1}^{4}\left(\kappa_{i}e^{i\cdot \Phi_{i}(x,y)}e^{ik\cdot R}\left(ik-\frac{1}{R}\right)\frac{z}{R^{2}}\right)$$

where $k=2\pi /\lambda_{0}$ is the wave number, *z* denotes the distance between target holographic focusing plane and exit‐facet of metasurface, $R=\sqrt{x^{2}+y^{2}+z^{2}}$ represents the relative distance between target focusing point and the specific meta‐atom in position (*x*, *y*), and the input wave can be expressed by the composition of orthogonal circular polarizations as $\left|{\vec{E}}_{\text{in}}\right\rangle =\kappa_{1\left(2\right)}\left|L\right\rangle +\kappa_{4\left(3\right)}\left|R\right\rangle$. Notably, the ratio of *κ*_1_/*κ*_4_ (or *κ*_2_/*κ*_3_) depends on the exploited input polarization state, while *κ*_1_/*κ*_2_ (or *κ*_4_/*κ*_3_) is relative to the energy ratio between co‐ and cross‐polarized components.

In order to invoke the quadruplex transmission coefficients simultaneously to synthesize a complete holographic imaging, two necessary requirements should be satisfied. One is the phase condition, i.e., fulfilling independent phase functions of ∠*T*_LL_, ∠*T*_LR_, ∠*T*_LR_, and ∠*T*_RR_, which guarantees the implementation of separate fractional holographic distributions. The other condition is the specific ratio *κ* between the four transmission intensities, as the ciphertext key that determines the matched arbitrary polarization state of the output wave and supports the quality and performance of resultant interference imaging. For a proof‐of‐concept demonstration, we set this ratio key as *κ*_*i*_ = 1 to maintain the output energy equally distributed, such that |*T*_LL_| ≈ |*T*_LR_| ≈ |*T*_RL_| ≈ |*T*_RR_|, and also to impose the integral holographic displaying in linear polarization.

Besides, to synchronously modulate quadruplex polarization channels in circular polarization base, the basic operation is to decouple the inherent consistence and conjugate symmetry between diagonal and off‐diagonal components in the transmission matrix *T*_cir_. Here, we introduce the chirality‐assisted phase combined with propagation and geometric phases.^[^ ^45^ ^]^ Chirality‐assisted phase is exploited to effectively decouple the consistency between the two co‐polarized CP channels (*T*_LL_ and *T*_RR_). Propagation phase can impose independent phase responses between co‐ and cross‐polarized channels (*T*_LL_ and *T*_RL_ or *T*_LR_ and *T*_RR_), whereas geometric phase can impose conjugate phase profiles in two cross‐polarized channels (*T*_LR_ and *T*_RL_). Notably, the inherent differences between chirality‐assisted phase and geometric phase include the target decoupling coefficients, and tailoring configuration parameters. It is also important to note that chirality‐assisted phase is related to the in‐plane pattern of the meta‐atom, while it is not the case for geometric phase.

### 2.2 Chirality‐Assisted Meta‐Atom Configuration

**Figure 2:**
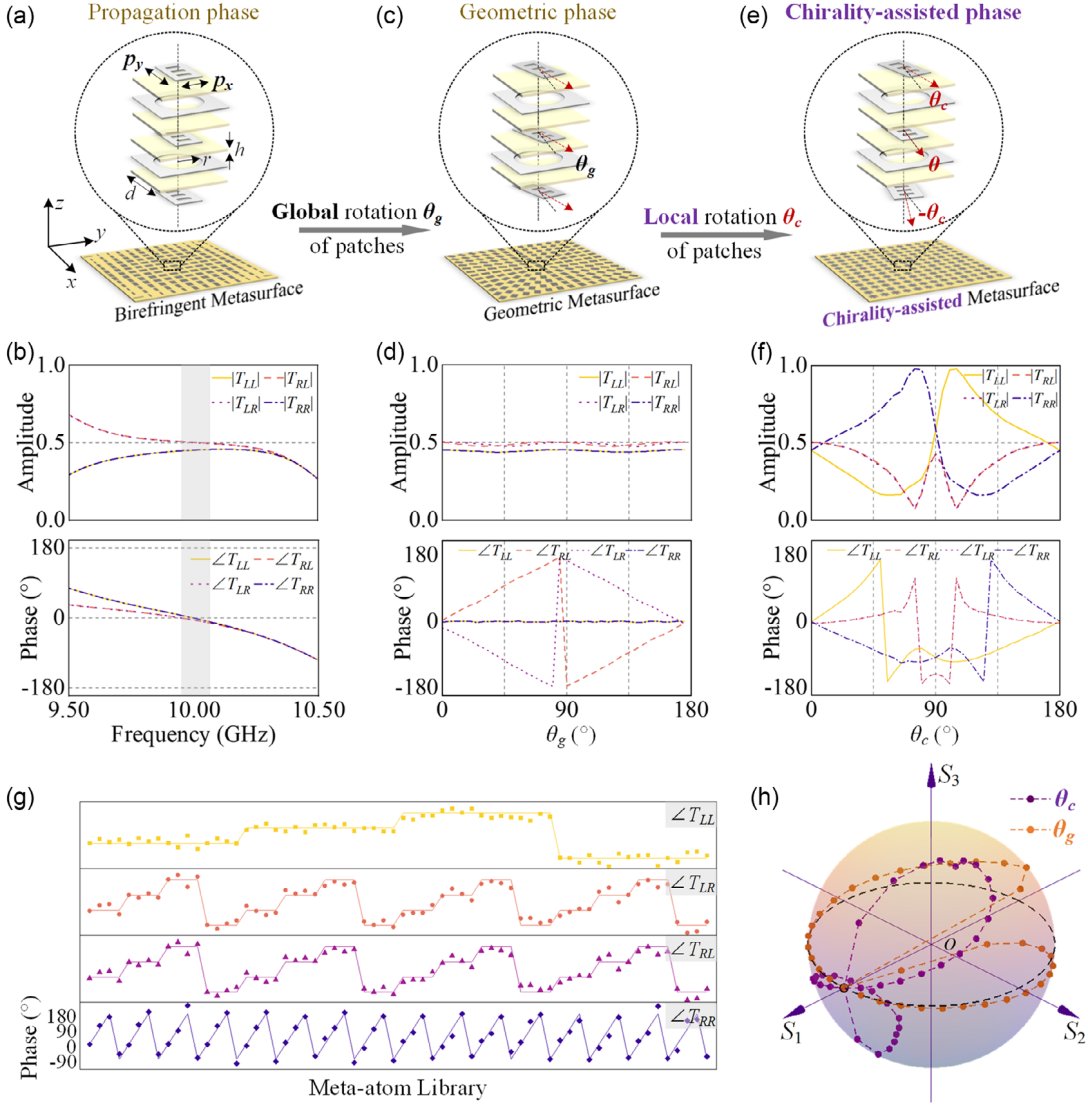
Meta‐atom configuration and demonstration. a) A simplified illustration of the birefringent metasurface, whose meta‐atom topology in the enlarged view is empowered with propagation phase. The periodicity is *d* = 10 mm, radius of circle aperture in grid mesh layer is *r* = 4.5 mm, thickness of each dielectric substrate is *h* = 1 mm, and thickness of metallic layers are 0.035 mm. The key factors for tailoring propagation phase are the width and length of the rectangular patches, which we set as *p*_*x*_ = 7.5 mm and *p*_*y*_ = 5.4 mm for *κ* = 1. b) Simulated quadruplex frequency profiles of this meta‐atom in circular polarization base for propagation phase verification. c) A simplified illustration of the geometric metasurface, where its meta‐atom configuration depicted in an enlarged view produces geometric phase by the global rotation angle *θ*_g_, and d) amplitude and phase responses against *θ*_g_ changing from 0° to 180°. e) A simplified illustration of chirality‐assisted metasurface, where its meta‐atom shown in an enlarged view produces the chirality‐assisted phase by the local rotation angle *θ*_c_, and f) amplitude and phase responses against *θ*_c_ changing from 0° to 180°. g) Quadruplex circularly polarized phase responses of meta‐atom library composed of 64 optimized configurations. h) Under linear polarized illumination, trajectories of output polarization states on Poincaré sphere against global and local rotation of meta‐atom.

Comparatively, concentration is focused on the integral effect of each circularly polarized component, determining that the key part is the execution of preset equal energy weight among intensity profiles. We exploit the stacking and twisted configurations that we have previously reported.^[^ ^42^ ^]^ The topological construction of the meta‐atom is shown in **Figure** **2**a, which is designed using five metallic layers, including three rectangular patch elements with parallel gaps and two grid mesh with circle aperture, interleaved by four dielectric substrates. This stacked meta‐atom is regarded as the multi‐port cascading network model, supporting the smooth filtering frequency bandwidth. Thereinto, three patch elements have irreplaceable roles in polarization selection and phase manipulation. Due to the geometric anisotropy feature from the rectangular patch element, the width *p*_*x*_ and length *p*_*y*_ separately tailor phase retardation and energy distribution ratio between co‐ (*T*_LL_, *T*_RR_) and cross‐polarized components (*T*_LR_, *T*_RL_).^[^ ^39^ ^]^

Here, we deliberately optimize a meta‐atom with the preset energy weight *κ* = 1 for demonstration, whose transmission profiles under illumination of both LHCP and RHCP waves are displayed in Figure 2b. The quadruplex amplitude responses of this meta‐atom are consistently maintained at 0.5 around 10 GHz (operation wavelength *λ*_0_ = 30 mm), and the corresponding primitive phases achieve ∠*T* (*f* = 10 GHz) = 0°. This resultant circularly polarized responses could advocate a basic model for further verifications. Then, we rotate the three patches around the normal axis, namely global rotation *θ*_g_ as illustrated in Figure 2c, to conduct the geometric phase of meta‐atom. Figure 2d presents the amplitude and phase responses in circular polarization base against *θ*_g_ at the operation frequency. The quadruplex amplitudes are insensitive to the global rotation, while the cross‐polarized ∠*T*_LR_ and ∠*T*_RL_ are conjugately imposed with phase interruptions 2*θ*_g_, agreeing well with the geometric phase scheme. Accordingly, we orientate the top and bottom patches clockwise and counterclockwise separately with the local rotation angle *θ*_c_, as shown in Figure 2e, to perform the chirality‐assisted phase effect. It can be seen from Figure 2f, the amplitude and phase profiles of two co‐polarization are obviously disunited from each other, and different decoupling effect is exhibited with *θ*_c_. The non‐linear decoupling function in the co‐polarized amplitude and phase profiles with respect to *θ*_c_ is triggered by the in‐plane anisotropic pattern and out‐plane interlaminated coupling. Even so, the two co‐polarizations can effectively achieve 2π phase coverage and the amplitude variations can be supplemented by judiciously adjusting the propagation phase.

Based on the above verification, we optimize 64 meta‐atom configurations to establish a library, and the ideal and simulated phase responses of such meta‐atom library are illustrated in Figure 2g. It can be observed that meta‐atoms in library can separately provide 2‐bit phase coverage of each circular polarization component with tolerable deviations. Meanwhile, the corresponding amplitudes of these 64 meta‐atoms are all optimized around 0.5, where the simulated average amplitude responses are |*T*_LL_|_aver_. = 0.579, |*T*_LR_|_aver_. = 0.503, |*T*_RL_|_aver_. = 0.502, and |*T*_RR_|_aver_. = 0.447, respectively. Additionally, Figure 2h depicts the corresponding output polarization states of a specific meta‐atom with variable rotation configurations on the Poincaré sphere. Under the illumination of *x*‐linearly polarized wave, the meta‐atom with global or local rotation can produce an output wave with arbitrary elliptical polarization states. Trajectories of output polarization states on the Poincaré sphere also indicate distinct transformation abilities of two different rotations, which also provides the foundation for further ciphering with complex elliptical polarizations.

## 3 Metasurface Construction and Performance Verifications

### 3.1 Metasurface Platform with Quadruplex Circular Polarization Modulations

**Figure 3:**
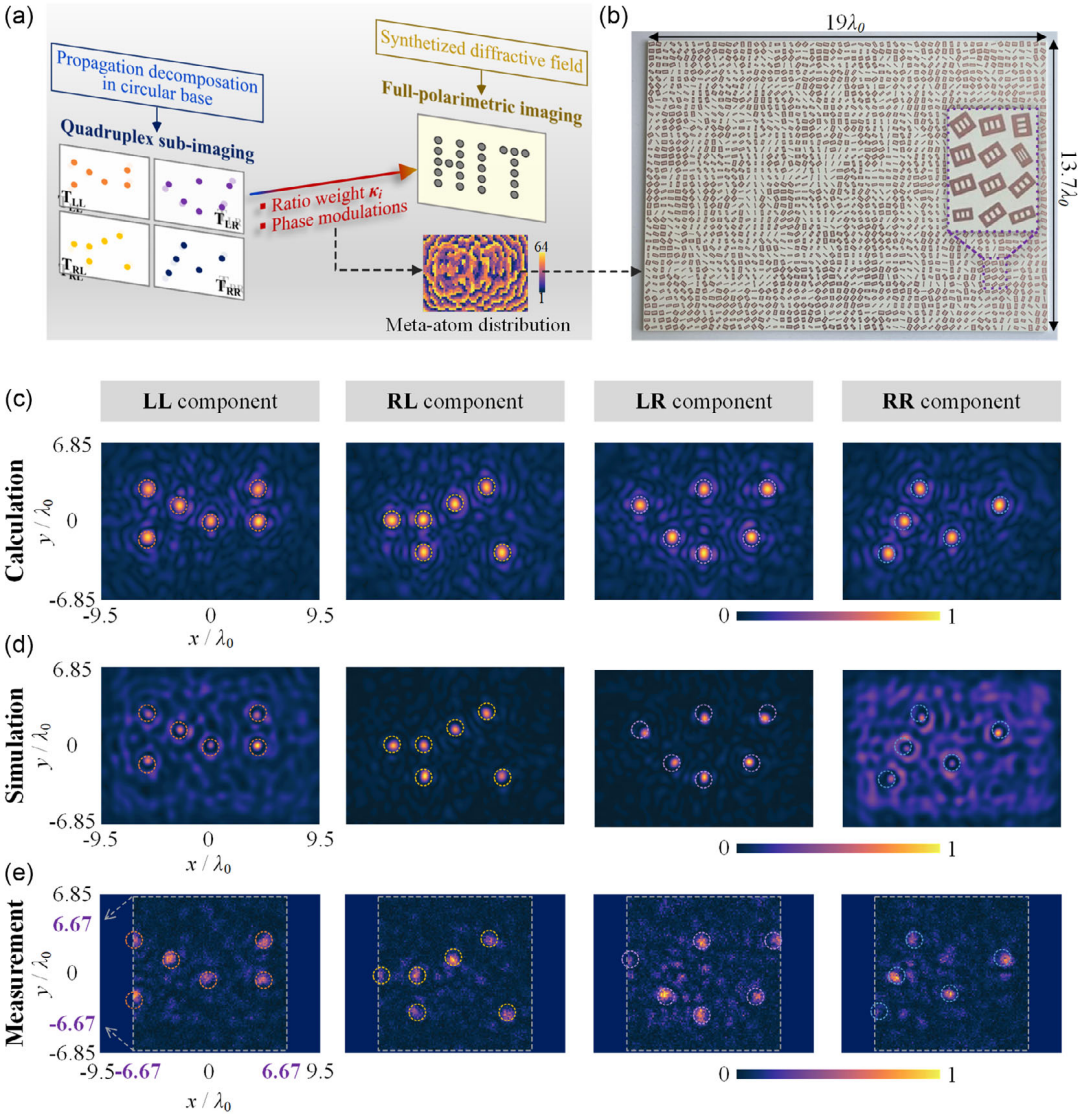
Demonstrations of constructed metasurface for quadruplex circular polarization wave‐front modulation. a) Schematic illustration of the synthetization of holographic “HIT” image, and the corresponding sub‐imaging in circularly polarized components, where the inset presents the theoretical metasurface construction with meta‐atoms picked from the library. b) Photograph of the fabricated metasurface platform in top view, and inset shows the zoomed view of several meta‐atom configurations. c) Theoretically calculated, d) numerically simulated, and e) experimentally measured intensity distributions of each circular polarization components *T*_LL_, *T*_LR_, *T*_RL_, and *T*_RR_, in *xy* plane at propagation distance *z* = 8.3*λ*_0_ in output transmission field at the operation frequency of 10 GHz.

The design principle of the full‐polarimetric synthesized hologram is based on the Gerchberg‐Saxton (GS) algorithm to obtain the spatial phase distributions of the target holographic displaying. Through inverse design of equivalent virtual source points, the adopted metasurface can provide point‐to‐point phase interruptions to support the constructive interferences of field intensity. Afterwards, the phase interruptions Ф at specific coordinates (*x*, *y*) on metasurface plane can be described by:^[^ ^26^ ^]^

(4)

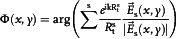

where *s* presents the number of the focusing points of the discretized target image, *t* is the number of meta‐atoms on the metasurface plane, ${\vec{E}}_{s}(x,y)$ represents the superimposed electric fields radiated from all *s* focusing points, and $R_{t}^{s}$ is the distance between *s*th focusing point and *t*th atom on metasurface plane with position (*x*, *y*). In this work, we preset the target imaging of “HIT”, which is characterized by 23 discretized focusing points for illustration (*s* = 23). To invoke the quadruplex polarization components, the “HIT” hologram is decomposed into four different sub‐sections, where each section containing 5–6 discretized points is achieved by *T*_LL_, *T*_LR_, *T*_RL_, and *T*_RR_, respectively. The holographic imaging plane is set as *f*_focal_ = 8.3*λ*_0_ with operation frequency 10 GHz. Under the principles of circular polarization decomposition and synthesized diffraction field, the quadruplex sub‐imaging and full‐polarimetric hologram is schematically illustrated in **Figure** **3**a.

As for metasurface platform construction, we pre‐suppose 57 × 41 meta‐atoms along *x*‐ and *y*‐axes, respectively, and the total geometric size is 19.0*λ*_0_ × 13.7*λ*_0_. According to these four preset sectional holograms, the required phase distribution functions are derived from Equation (4), and the theoretical metasurface construction with the corresponding meta‐atoms from the library is displayed in the inset of Figure 3a. To further experimentally verify the effectiveness of the proposed full‐polarimetric synthetization scheme, the metasurface is fabricated by exploiting classical printed circuit board (PCB) technique. Figure 3b presents a photograph of the fabricated metasurface sample, and inset shows a zoomed top view of several meta‐atoms.

First, we exhibit the theoretically calculated intensity distributions of the quadruplex polarization components in Figure 3c. The dashed circles label the specific positions of target focusing points. The required holographic distributions with several discretized foci of four polarization components are all obtained from Equation (3). It is worth noting that, although there theoretically exist inevitable diffraction interferences around discretized foci, the holographic sub‐imaging can be still clearly distinguished in the detection regions. Based on full‐wave simulations, the proposed metasurface platform can effectively generate distinct hologram sub‐imaging in co‐and cross‐polarized transmission fields under LHCP and RHCP illuminations, respectively. The output intensities of each polarization components are displayed in Figure 3d, where the distribution tendencies are in accordance with the theoretical predictions. Experimental measurements have also been conducted in the microwave region (the setups and procedures are stated in Experimental Section). Figure 3e represents the corresponding measured intensity distributions of |*T*_LL_|, |*T*_LR_|, |*T*_RL_|, and |*T*_RR_|. Due to the limited scanning range of measurement setup, we can only exhibit detection region with $x\in \left[-6.67\lambda_{0},6.67\lambda_{0}\right]$ and $y\in \left[-6.67\lambda_{0},6.67\lambda_{0}\right]$, which is highlighted by the white dashed rectangle. We can obviously observe that the sub‐imaging focusing points in measurements agree well with the calculations. The measured resolution of the focusing points is 1.064λ_0_/NA, which can be considered as high‐resolution imaging according to the Airy spot size of 1.22λ_0_/NA. Although the measured intensities are deteriorated to some extent, which can be attributed to the quasi‐plane (spherical) wave illumination of illuminating horn antenna source, these results effectively indicate the feasibility of the chirality‐assisted phase scheme and separate modulation abilities of quadruplex circular polarization components.

### 3.2 Synthesized Integral Hologram Displaying under Linear Polarization

**Figure 4:**
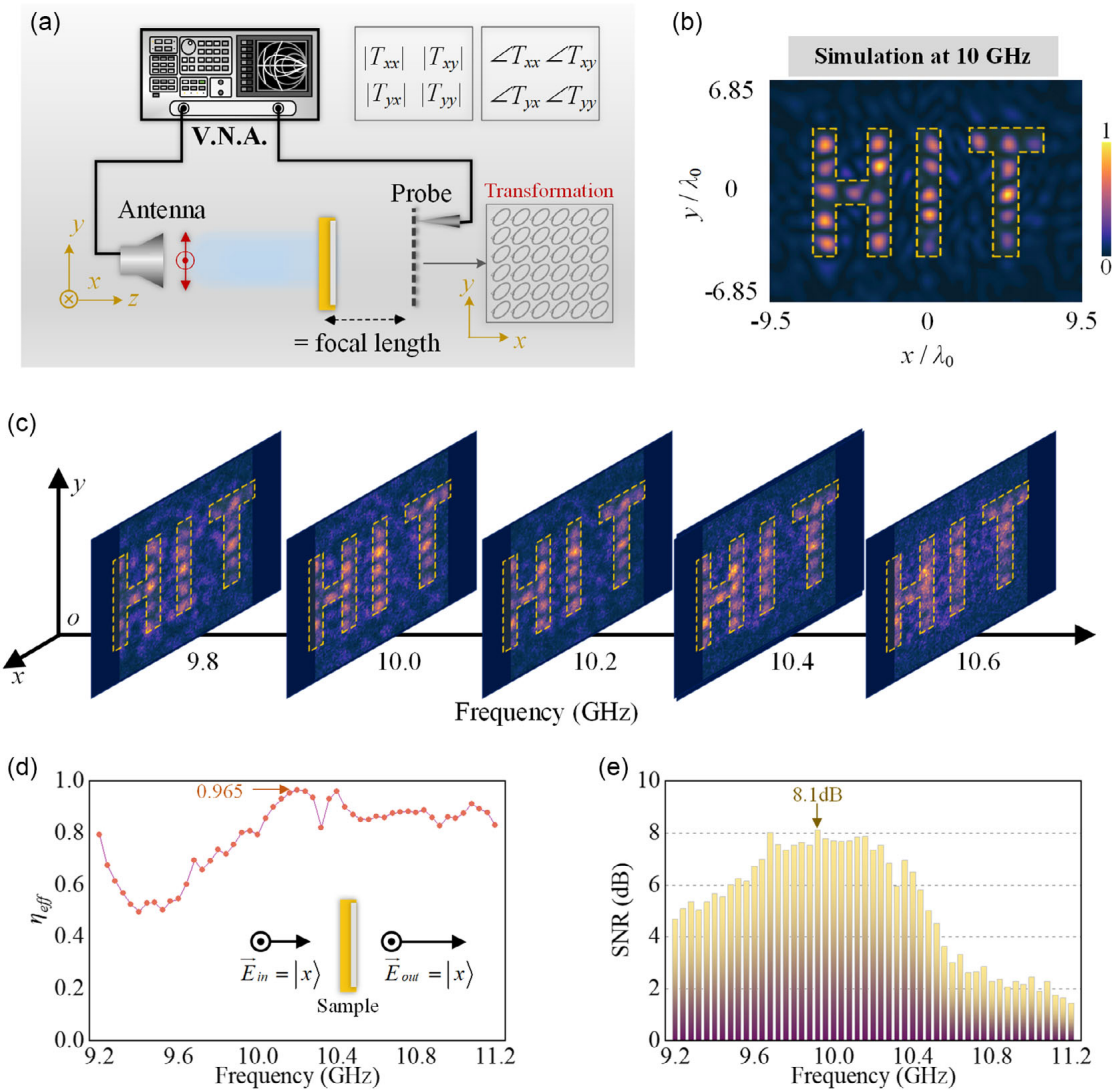
Synthesized integral hologram displaying under linear polarization. a) Schematic of near‐field measurement setup and polarization calculation procedure. b) Simulated linearly polarized intensity distribution of holographic “HIT” imaging in *xy* plane at propagation distance *z* = 8.3*λ*_0_ under linear polarized illumination. c) Measured linearly polarized holographic “HIT” distributions in *xy* plane at sampling frequency points 9.8, 10.0, 10.2, 10.4, 10.6 GHz, respectively, where the dashed zone illustrates the preset target holographic region. d) Measured efficiency and e) SNR of the proposed metasurface sample within 9.2–11.2 GHz under a linearly polarized incident wave. The inset in (d) presents the schematic process of linear polarized wave passing through the metasurface sample.

According to the synthesized diffraction field theory in Equation (3), we further aim to assess the performances of integral holographic displaying based on the above demonstration of sub‐imaging in circular polarization components. **Figure** **4**a illustrates the schematic of full‐polarimetric synthetization method. As illustrated in Experimental Section, the fabricated sample is illuminated by transmitting antenna with horizontally and vertically polarized electric field, while the receiving end is a dielectric probe oriented horizontally and vertically to measure the linearly polarized fields ${\vec{E}}_{x}$ and ${\vec{E}}_{y}$. Thus, the complex transmission coefficients in linear polarization base, $T_{\text{lin}}=\left[\begin{array}{cc} T_{xx} & T_{xy}\\ T_{yx} & T_{yy} \end{array}\right]$ with specific amplitude and phase responses, can be measured via the vector network analyzer (VNA). Based on this measured transmission matrix of the proposed metasurface, the practical output performances under any arbitrary polarized incident waves can be transformed and visualized. The intensity ratio is set as *κ*_*i*_ = 1, resulting in a hologram under linear polarization state. Under an *x*‐linearly polarized incident wave, the proposed metasurface platform can generate distinguish “HIT” holographic image in transmitted *xy* plane with propagation distance *z* = 8.3*λ*_0_, and the corresponding simulated intensity distribution with linear polarization at operation frequency 10 GHz is displayed in Figure 4b. The unambiguous 23 focusing points constitute the hologram target “HIT”, indicating that each sub‐imaging of circular polarization equally contributes to the reconstruction of the hologram.

Accordingly, the measured output intensity distributions in *xy* plane at five frequency points under *x*‐linearly polarized illumination with interval sampling from 9.8 to 10.6 GHz are exhibited in Figure 4c. The targeted HIT image can be observed at different frequencies, and the optimal result appears at 10.2 GHz. This slight deviation of operation frequency is primarily attributed to the fabrication error, which would affect the practical feasibility of the proposed full‐polarimetric synthetization scheme for holographic displaying. In order to further evaluate the holographic performances of the fabricated metasurface prototype, two feature parameters are introduced, including the overall efficiency and the signal‐to‐noise ratio (SNR). Firstly, the overall efficiency *η*_eff_ is defined as the ratio between the output energy distributed in target polarized fields to the energy of input wave, as expressed by:
(5)

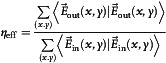

where 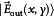 and 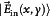 express the output and input electric fields with linear polarization states at specific coordinates (*x*, *y*) in the measured holographic plane. Hence, *η*_eff_ characterizes the transmission performance and polarization transformation capabilities of the proposed metasurface. The measured efficiency can reach as high as 80% from 10 to 11 GHz, with a maximum approaching 96.5% at 10.2 GHz, as displayed in Figure 4d. Such high efficiency performance indicates that the transmitted holographic display with specific polarization states can well match the input linear polarization. We shall note that there exists serious energy loss around 9.4 GHz, which results from mismatching conditions in transmission mode, with almost half of the incident energy is reflected. In contrast, SNR is introduced to evaluate the imaging quality of the proposed holographic displaying scheme, where the signal is regarded as the electric intensity in target HIT region (${\mathbb{Z}}_{\text{HIT}}$) labeled by dashed box and noise is deemed as that out of ${\mathbb{Z}}_{\text{HIT}}$. Thus, SNR parameter is calculated by:

(6) $$\text{SNR}=20\mathrm{lg}\frac{\sum_{(x,y)\subset {\mathbb{Z}}_{HIT}}\left|\left|{\vec{E}}_{\text{signal}}(x,y)\right\rangle \right|}{\sum_{(x,y)⊄{\mathbb{Z}}_{HIT}}\left|\left|{\vec{E}}_{\text{noise}}(x,y)\right\rangle \right|}$$

where $\left|{\vec{E}}_{\text{signal}}(x,y)\right\rangle$ and $\left|{\vec{E}}_{\text{noise}}(x,y)\right\rangle$ denote the signal and noise electric intensities at position (*x*, *y*) in transmission field. Figure 4e illustrates the measured SNR within bandwidth 9.2–11.2 GHz, and the optimal SNR performance appears coincidently with the overall efficiency around operation frequency 10 GHz. Although this SNR result presents operation frequency deviation, the high performance of measured hologram quality is verified, indicating the successful synthetization of the target imaging.

### 3.3 Performance Verification under Arbitrary Elliptical Polarizations

**Figure 5:**
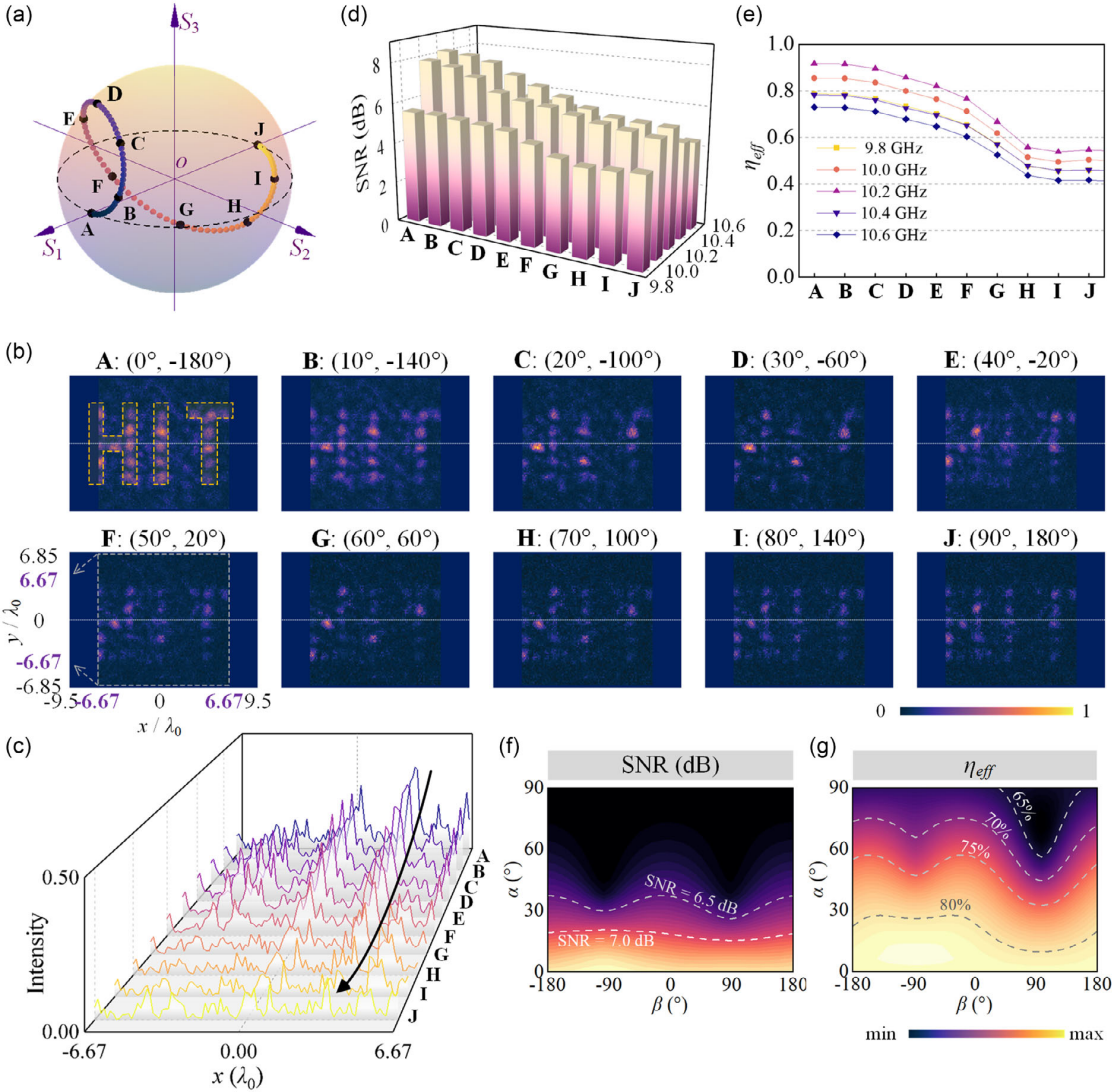
Performance verification under arbitrary elliptically polarized illumination. a) Schematic of incident polarization states sampling, as labelled by **A–J** along trajectory path on the Poincaré sphere. b) Measured intensity distribution of holographic “HIT” imaging in preset focal plane under incident wave with variable polarization states from **A** to **J**. c) Normalized intensity profiles along *y* = 0 axis extracted from the white dotted lines in b). d) SNR and e) efficiency performances of metasurface sample under **A** to **J** polarization states illumination at sampling frequency points 9.8, 10.0, 10.2, 10.4, 10.6 GHz. Mappings of experimental f) SNR and g) efficiency of metasurface sample against full‐stokes polarizations at 10.2 GHz.

For the verification of the performances, it is necessary to evaluate the robustness and sensitivity characteristics of the proposed full‐polarimetric synthetized metasurface to arbitrary elliptical polarizations. Here, we exploit arbitrary elliptical polarization |α,β⟩ as illumination source states, and the corresponding output intensities are to be observed in holographic planes. Initially, 10 typical elliptical polarizations are randomly selected along the trajectory path on the Poincaré sphere shown in **Figure** **5**a, whose Jones vector angles α∈[0°,90°] and β∈[−180°,180°] with equally sampling interval Δ(*α*, *β*) = (10°, 40°) are labelled from “**A**” to “**J**”. Based on the measured transmission coefficients of the fabricated metasurface platform, when the polarization states of illumination source are set as 10 elliptical polarization states, respectively, the output fields can be obtained by:

(7) $$\left|{\vec{E}}_{\text{out}}\left(x,y\right)\right\rangle =T_{\text{lin}}^{\text{mea}}\left(x,y\right)\left|\alpha ,\beta \right\rangle =\left[\begin{array}{c} T_{xx}\left(x,y\right)\cos\alpha +T_{xy}\left(x,y\right)\sin\alpha e^{i\beta }\\ T_{yx}\left(x,y\right)\cos\alpha +T_{yy}\left(x,y\right)\sin\alpha e^{i\beta } \end{array}\right]$$

Accordingly, the intensity distribution of output fields $\left|\left|{\vec{E}}_{\text{out}}\left(x,y\right)\right\rangle \right|$ are displayed in Figure 5b. With the variation of the input elliptical polarization state, the output intensity fields exhibit obviously distinct tendencies. For some input polarizations, it is even difficult to recognize the synthesized holographic HIT imaging, revealing that the proposed full‐polarimetric holography scheme is quite sensitive to the input polarization state. This polarization‐dependent property guarantees the scheme feasibility of synthetization weight *κ* of quadruplex polarizations as the ciphertext key. The normalized intensity profiles along center axis *y* = 0 are extracted and compared in the holographic plane of all these ten output fields, as presented in Figure 5c and labelled by white dotted lines in Figure 5b. It is seen that there exist several peaks in each intensity curves within *x*‐axis range of [−6.67*λ*_0_, 6.67*λ*_0_], illustrating the corresponding focusing points located along *x*‐axis. Through changing the input polarization from **A** to **J**, the intensity peaks of the focusing points are gradually reduced, which implies the decayed imaging quality when the input polarization is mismatched. Figure 5d,e present the calculated SNR and *η*_eff_ properties of the output holographic HIT imaging under variable illuminating elliptical polarizations at 9.8, 10.0, 10.2, 10.4, and 10.6 GHz. The maximum SNR is 8.1 dB and *η*_eff_ is 96% of output holographic imaging appear under the illumination state **A** at 10.2 GHz, and the minimum performances are obtained under polarization state **J**.

Moreover, we also take SNR and *η*_eff_ as two evaluation indicators to calculate influence of illumination polarization with ergodicity of all possible states. Figure 5f displays the measured SNR and *η*_eff_ of output transmission fields at 10.2 GHz, mapping to incident wave carrying full‐stokes polarizations. We observe that when the input polarization approaching the target ciphertext weight *κ*, i.e., *x*‐linear polarization, the optimal imaging quality and efficiency can be obtained. On the contrary, with the input polarization changing to the orthogonal *y*‐linear state, SNR performance degrades and the efficiency decreases. Meanwhile, the vector angle *β*, characterizing phase retardation between two vector components, would produce less influence on the holographic performances. These results under elliptical polarization sources can provide solid foundation for future applications based on the elliptical polarization imposed ciphertext holographic displaying.

## 4 Conclusion

To summarize, we have presented a full‐polarimetric synthetization scheme based on synchronous modulation of quadruplex coefficients in complex transmission matrix. Chirality‐assisted phase is adopted with propagation and geometric phases to decouple the inherent consistency and conjugate symmetricity between four circular polarization components, which provides the implementation foundation for synthetization holography. Through pre‐setting linear polarization as the cipher‐state for integral holographic imaging generation, the output intensity of “HIT” can be successfully produced in transmission field under the matched cipher input state. High performance of working efficiency (>90%) and SNR (>7 dB) are experimentally achieved around the operation frequency. These results can effectively prove the feasibility of our proposed synthetization scheme for full‐polarimetric holographic displaying. Besides, we have also verified the sensitivity and robustness of full‐polarimetric hologram imaging against variable input polarization states. Experimental measurements demonstrate that the target integral hologram “HIT” exhibit optimal performances, including distinguishability, SNR, and efficiency, under the cipher input linear polarization state. Such demonstrations prove the feasibility of hologram encryption approaches taking arbitrary elliptical polarizations as ciphertexts, can offer extra degree of freedom and novel solutions for further cryptography with high‐level security.

## 5 Experimental Section

5.1

5.1.1

##### Fabrication Method

The prototype is fabricated by classical printed circuit board (PCB) technique. Taking into account the multi‐layer configuration of the sample, thermocompression bonding technique is utilized to assemble each treated copper‐covered layer with placing adhesive layers. For aligning the layers, cameras with imaging systems are used and the layers can be positioned to fit properly if imprecision is detected. The different layers are then stacked and hot pressed with bonding prepreg layers. In the fabrication process, the sample is composed of four pieces of polyfluortetraethylene dielectric‐slabs and three adhesive layers. The 1 mm thick dielectric substrate used has a relative permittivity *ε*_r_ = 3.5 and double copper‐cladding layer of 0.035 mm thick, while the adhesive layer has a relative permittivity *ε*_r_ = 2.74 and thickness of 0.1 mm. The total thickness of fabricated samples is about 0.15*λ*_0_ at 10 GHz and the proposed metasurface consists of 57 × 41 polyatomic units with equivalent total size of 570 mm × 410 mm.

##### Experimental Setups

Near‐field scanning is conducted by a setup in an anechoic chamber. In the measurement process, a vector network analyzer (VNA) is used to record the complex transmitted field information (*S*_21_ parameter). A 2–18 GHz dual linearly polarized wideband horn antenna is used as the feeding source, and a hybrid coupler is inserted to impose ± 90° phase differences between the linearly polarized inputs to generate LHCP or RHCP waves. The distance relative to metasurface is set to more than 40λ_0_ for quasi‐plane wave illumination. For near‐field sensing, a fiber optic active antenna is used as a field probe to measure both the amplitude and phase of the electric field. This purely dielectric probe has an ultrasmall head of 6.6 × 6.6 mm^2^, owning portability and movable abilities, and negligible field perturbation.^[^ ^48^ ^]^ The field‐sensing probe is fixed on two translation stages controlled by a motion controller and its position is incremented by a step of 2 mm. Hence, we are able to collect the output electric field information covering the whole *xy* detection plane at a specific propagation distance *z*. The region of detection is about 13.3*λ*_0_ × 13.3*λ*_0_, at focal distance *z* = 8.3*λ*_0_. Notably, the probe can be used along horizontal and vertical directions to collect *x*‐ and *y*‐linearly polarized fields, i.e., *E*_*x*_ and *E*_*y*_ components. Transmission electric fields for left‐ and right‐handed circular polarizations is obtained simply using the expressions *E*_L_ = *E*_*x*_ + *iE*_*y*_ and *E*_R_ = *E*_*x*_ − *iE*_*y*_, respectively. Through combining the complex transmitted field information under different polarization states of the illuminating antenna, quadruplex transmission field components in the circular polarization base can be measured, including *L*‐output under *L*‐input, termed as complex component *T*_LL_, the *R*‐output under *L*‐input as *T*_RL_, the *L*‐output under *R*‐input as *T*_LR_, and the *R*‐output under *R*‐input as *T*_RR_. Then, the complex electric distribution in the whole imaging region can be obtained, including quadruplex circularly polarized transmission channels.

## Conflict of Interest

The authors declare no conflict of interest.

## Data Availability

The data that support the findings of this study are available from the corresponding author upon reasonable request.
